# Multiomic molecular patterns of lipid dysregulation in a subphenotype of sepsis with higher shock incidence and mortality

**DOI:** 10.1186/s13054-024-05216-3

**Published:** 2024-12-24

**Authors:** Beulah Augustin, Dongyuan Wu, Lauren Page Black, Andrew Bertrand, Dawoud Sulaiman, Charlotte Hopson, Vinitha Jacob, Jordan A. Shavit, Daniel A. Hofmaenner, Guillaume Labilloy, Leslie Smith, Emilio Cagmat, Kiley Graim, Susmita Datta, Srinivasa T. Reddy, Faheem W. Guirgis

**Affiliations:** 1 Department of Emergency Medicine, University of Florida College of Medicine, 1329 SW 16thStreet, Gainesville, FL 32610 USA; 2 Department of Biostatistics, University of Florida, Gainesville, FL USA; 3 Department of Emergency Medicine, Northwestern University Feinberg School of Medicine, Chicago, IL USA; 4 Division of Cardiology, Department of Medicine, David Geffen School of Medicine at UCLA, Los Angeles, CA 90095 USA; 5 Department of Emergency Medicine, University of Michigan Medical School, Ann Arbor, MI USA; 6 Department of Pediatrics, University of Michigan Medical School, Ann Arbor, MI USA; 7 Department of Human Genetics, University of Michigan School of Medicine, Ann Arbor, MI USA; 8 Institute of Intensive Care Medicine, University Hospital Zurich, Raemistrasse 100, CH-8091 Zurich, Switzerland; 9 UF Health Jacksonville Center for Data Solutions, Jacksonville, FL USA; 10 Computer and Information Science Engineering, College of Engineering, University of Florida, Gainesville, FL USA; 11 UF Health Cancer Center, UF Genetics Institute, Gainesville, FL USA

**Keywords:** Sepsis, Cholesterol, Lipids, Phenotyping

## Abstract

**Background:**

Lipids play a critical role in defense against sepsis. We sought to investigate gene expression and lipidomic patterns of lipid dysregulation in sepsis.

**Methods:**

Data from four adult sepsis studies were analyzed and findings were investigated in two external datasets. Previously characterized lipid dysregulation subphenotypes of hypolipoprotein (HYPO; low lipoproteins, increased mortality) and normolipoprotein (NORMO; higher lipoproteins, lower mortality) were studied. Leukocytes collected within 24 h of sepsis underwent RNA sequencing (RNAseq) and shotgun plasma lipidomics was performed.

**Results:**

Of 288 included patients, 43% were HYPO and 57% were NORMO. HYPO patients exhibited higher median SOFA scores (9 vs 5, *p* = < 0.001), vasopressor use (67% vs 34%, *p* = < 0.001), and 28-day mortality (30% vs 16%, *p* = 0.004). Leukocyte RNAseq identified seven upregulated lipid metabolism genes in HYPO (*PCSK9, DHCR7, LDLR, ALOX5, PLTP, FDFT1*, and *MSMO1*) vs. NORMO patients. Lipidomics revealed lower cholesterol esters (CE, adjusted *p* = < 0.001), lysophosphatidylcholines (LPC, adjusted *p* = 0.001), and sphingomyelins (SM, adjusted *p* = < 0.001) in HYPO patients. In HYPO patients, *DHCR7* expression strongly correlated with reductions in CE, LPC, and SM (*p* < 0.01), while *PCSK9, MSMO1, DHCR7, PLTP,* and *LDLR* upregulation were correlated with low LPC (*p* < 0.05). *DHCR7, ALOX5*, and *LDLR* correlated with reductions in SM (*p* < 0.05). Mortality and phenotype comparisons in two external datasets (N = 824 combined patients) corroborated six of the seven upregulated lipid genes (*PCSK9, DHCR7, ALOX5, PLTP, LDLR,* and *MSMO1*).

**Conclusion:**

We identified a genetic lipid dysregulation signature characterized by seven lipid metabolism genes. Five genes in HYPO sepsis patients most strongly correlated with low CE, LPC, and SMs that mediate cholesterol storage and innate immunity.

**Supplementary Information:**

The online version contains supplementary material available at 10.1186/s13054-024-05216-3.

## Introduction

Sepsis is a potentially deadly condition stemming from a dysregulated systemic response to infection [1]. Lipoprotein cholesterols are highly biologically active in sepsis [2]. The dysregulated host response to sepsis also leads to dysregulation in lipid metabolism, with implications on host defense, inflammation, and pro-oxidant lipids [2–5]. Lipoprotein cholesterols are generally thought to be protective in sepsis with pleiotropic effects, immune defense properties, and the ability to transport toxins out of circulation [6]. However, in sepsis their levels fall dramatically, with the degree of drop in cholesterol levels being predictive of organ failure and ICU mortality [7–9].

Lipidomic and genetic studies have recently shed light on lipid metabolic dysregulation in sepsis [10–16]. Chouchane et al. observed a significant reduction in plasma lipid levels among community-acquired pneumonia (CAP) patients with sepsis [10]. Specifically, cholesterol esters and lysophospholipids were reduced in CAP sepsis patients, while triacylglycerols were elevated. We recently described the lipidomic profiles of our sepsis patients by clinical outcomes of chronic critical illness (CCI), early death, or rapid recovery [17]. In CCI/early death sepsis patients, we observed reductions in fatty acid (FA) 12:0 but elevations in FA 17:0 and 20:1 compared to rapid recovery. We also identified elevations in pro-inflammatory lipids including 15-hydroxyeicosatetraenoic (HETE), 12-HETE, and 11-HETE (oxidation products of arachidonic acid) and the pro-resolving lipid mediator, 14(S)-hydroxy docosahexaenoic acid (14S-HDHA) in CCI or early death sepsis patients compared to rapid recovery. The importance of specific lipid and cholesterol metabolism genes including *PCSK9, ALOX5*, *CETP, and DHCR7* in sepsis have been observed [11–16]. In most cases, upregulation of these genes was associated with increased mortality.

To better understand lipid dysregulation in sepsis and its contribution to heterogeneity, we previously described two sepsis subphenotypes based on lipoproteins and clinical profiles [18]. Hypolipoprotein (HYPO) patients were found to have lower cholesterol and lipoprotein levels (high density lipoprotein cholesterol, ApoA-I, PON1), increased endothelial cell dysfunction, and increased mortality and organ failure compared to normolipoprotein (NORMO) subphenotype sepsis patients. To better understand lipid metabolic dysregulation in sepsis, we sought to study gene expression and lipidomic patterns of these clinical subphenotypes to potentially identify pathways and targets for precision medicine. Our primary objective was to compare the transcriptomic and lipidomic profiles of HYPO vs. NORMO sepsis patients to gain a better understanding of lipid metabolic dysregulation in sepsis.

## Methods

### Study design and patient recruitment and enrollment procedures

We analyzed samples and data from three observational studies and one clinical trial of a lipid emulsion, plus additional samples from our research data and tissue bank [17–21]. For the clinical trial, only pre-drug patient samples and data were utilized for this analysis and thus the clinical trial drug did not have any effects on cholesterol levels, organ function, or mortality. All sepsis study patients were treated in the emergency department (ED) at UF Health Jacksonville between November 2016 and July 2022. The “Supplemental Flow Diagram” displays relevant studies and analyses performed on each study for reference. Ethical approval for all human studies was obtained from the University of Florida Institutional Review Board (IRB-01, valid until 01/06/2026), and the studies were registered on clinicaltrials.gov (NCT02934997; NCT04576819; NCT03405870). Adherence to the STROBE guidelines for observational studies was maintained throughout the analysis [22]. Trained research coordinators or providers identified emergency department patients who met Sepsis-3 criteria within 24 h of diagnosis. Patient recruitment occurred 7 days per week. Exclusion criteria were: (a) significant traumatic brain injury (evidence of neurologic injury on CT scan and a GCS < 8), (b) refractory shock (likely death within 12 h), (c) alternative or confounding diagnosis causing shock, (d) uncontrollable source of sepsis, (e) patients deemed futile care, (f) severe CHF (NY Heart Association Class IV), (g) Child–Pugh Class B or C liver disease, (h) known HIV with CD4 count < 200 cells/mm3, (i) absolute neutrophil count < 500 cells/mm^3^, (j) organ transplant recipient on immunosuppressive agents, (k) known pregnancy, (l) inability to obtain informed consent, and (m) diagnosed disorders of lipid metabolism.

### Data collection and adjudication

Clinical and laboratory data were collected by trained research coordinators and entered into a Research Electronic Data Capture (REDCap) database [23]. Collected data included demographics, sources of infection, comorbidities, vital signs, sequential organ failure assessment (SOFA) scores, antibiotic timing, fluid volumes, vasopressor and mechanical ventilation use, hospital and ICU lengths of stay, and 28 and 90-day mortality. Clinical diagnoses, outcomes, infection sources, culture results, and hospital dispositions underwent group adjudication by at least two clinician-investigators. Mortality for patients lost to follow-up was determined using the Social Security Death Index.

### Blood sampling, RNA sequencing, and RT-qPCR analysis

Blood samples were collected at enrollment and within 24 h of sepsis recognition and before any drug administration for one clinical trial. Clinical laboratory tests included lipid levels (total cholesterol, high density lipoprotein (HDL), low density lipoprotein (LDL), triglycerides) and SOFA score parameters. RNA sequencing (RNAseq) was performed using the Illumina NextSeq 550 system, while RT-qPCR analysis utilized Bio-Rad iQ SYBR Green Supermix.

### Shotgun lipidomics/lipid panel

The process of lipid extraction for shotgun lipidomic analysis has been detailed elsewhere [17]. 25 μl of plasma was extracted using a modified Bligh and Dyer method, with initial and final ratios of 0.9:2:1 and 1.9:2:1.9 (water:methanol:chloroform). Samples were spiked with 70 lipid standards across 17 subclasses before extraction. Pooled organic layers from two extractions were dried in a Thermo SpeedVac, resuspended in 300 μl of 1:1 methanol/dichloromethane with 10 mM Ammonium Acetate, and transferred to robovials for analysis. Samples were analyzed on the Sciex 5500™ with DMS device, targeting 1450 lipid species across 17 subclasses. Data acquisition and analysis were performed in Analyst 1.7.1 and Shotgun Lipidomics Assistant. The 1450 lipid species were acquired over two 75 μl infusions, with each MRM acquired 20 times. Raw signals were quantified against standards and normalized to plasma volume.

The LC–MS/MS method covers 39 bioactive lipids and pathway markers from cyclooxygenase and lipoxygenase products. Each analyte was paired with one of 19 deuterated internal standards or a structurally similar one co-eluting within 0.5 min. Details of the methods are provided in the “Supplemental Tables and Methods” section.

#### Unsupervised clustering analysis for patient classification

We analyzed data from each prospective study using 15 features identified previously [18]. As in our original study, we scaled the data, capped outliers to ± 3 standard deviations, and computed linkage matrices using Spearman’s correlation and Ward’s Method [18, 24, 25]. From the linkage matrices, which were computed using unsupervised hierarchical agglomerative clustering, we reconfirmed that the first two clusters were well segregated and extracted the patient populations as we did in our previous study [18]. Descriptive statistics for each cluster were overall consistent with subphenotype differences observed previously, and showed that one cluster had lower cholesterol levels (HDL-C, LDL-C, and total cholesterol) and higher total SOFA, and was overall consistent with the HYPO subphenotype ("Hypolipoprotein cluster"), while the other cluster was consistent with the NORMO subphenotype ("Normolipoprotein cluster"). Statistical comparisons between subphenotypes for continuous variables were calculated using Wilcoxon Rank-Sum tests and comparisons for categorical variables were calculated using Chi-Squared tests.

### Data analysis

Transcriptomic and lipidomic data analysis was conducted using R version 4.3.0 (R Core Team 2023, Vienna, Austria). Sequencing reads were aligned to the hg38 genome (GRCh38.p11) using STAR (v.2.7.9a) and featureCounts (v.2.0.3) [26, 27]. Subsequently, we performed differential expression analysis using DESeq2 with a negative binomial generalized linear model, adjusting for batch effects [28]. Differentially expressed genes were identified based on adjusted *p*-values obtained through false discovery rate (FDR) correction using the Benjamini–Hochberg method, with a significance threshold set at less than 0.05 [29]. We focused on a set of 47 a priori lipid metabolism genes [16]. To explore the importance of significant lipid genes, we employed a Random Forest with the default settings to predict HYPO vs. NORMO based on these genes [30].

To further investigate transcriptomic findings, we obtained publicly available microarray data from a study by Scicluna et al. from Refine.Bio [31] and an RNAseq study by Baghela et al. from the Sequence Read Archive. [32, 33] Transcripts were quantified using Salmon [34] and tximport [35]. We performed differential expression analysis using limma [36] for microarray data and DESeq2 [28] for RNAseq data. To correct for multiple testing, we applied the Benjamini–Hochberg method to control the FDR across all genes. Correlation analysis was conducted using the Pearson correlation coefficient.

Lipidomics and lipid panel data were analyzed using two-sample t-tests with FDR correction via the Benjamini–Hochberg method [27]. For shotgun lipidomics, we analyzed 355 lipids after filtering out those with missing values. For the lipid panel, we retained 7 lipids with at least 85% completeness, using the R package mice for multiple imputations on missing data [29]. This process created 5 imputed datasets with 10 iterations each. Adjusted p-values were aggregated using the Cauchy combination rule [30]. For survival analysis, we compared HYPO and NORMO subphenotypes using a log-rank test.

## Results

Data from 288 prospectively enrolled sepsis patients were analyzed. The median age was 63 years (IQR 56–72.9) and 53% of participants were male. Among enrolled patients, 51% were Black and 46% were White. The most common comorbidities were diabetes mellitus, chronic obstructive pulmonary disease, and end-stage renal disease. Median total cholesterol, HDL-C, and LDL-C levels for the whole cohort were 91.6 (IQR 74–122), 26 (IQR 15–38), and 40.4 (IQR 26.0–61.0) mg/dL, respectively. The median SOFA score for the overall cohort was 7 (IQR 4–10), and nearly half of all patients were mechanically ventilated, while over one-third required vasopressors. Twenty-eight-day mortality was 22%. Demographics, cholesterol levels, and clinical features are presented in Table 1**.** Sources of infection are presented in Supplemental Table 1.

**Table 1:** Clinical features and cholesterol levels

	Total cohort (n = 288)	HYPO cohort (n = 125)	NORMO cohort (n = 163)	*p*
Age (median [IQR])	63.0 [56.0, 72.9]	64.0 [56.4, 73.0]	63.0 [55.3, 71.0]	0.534
Sex (n, %)				
Male	154 (53%)	62 (50%)	92 (56%)	0.249
Female				
Race (n, %)				
Black	148 (51%)	62 (50%)	86 (53%)	0.862
White	133 (46%)	60 (48%)	73 (45%)	
Other	7 (2%)	3 (2%)	4 (2%)	
Comorbidities				
COPD (n, %)	54 (19%)	23 (18%)	31 (19%)	0.894
Diabetes (n, %)	113 (39%)	49 (39%)	64 (39%)	0.991
ESRD (n, %) (42 missing)	29 (10%)	13 (10%)	16 (10%)	0.804
Cancer (n, %)	22 (8%)	6 (5%)	16 (10%)	0.112
HIV (n, %)	9 (3%)	3 (2%)	6 (4%)	0.536
Statin use (n, %)	110 (38%)	45 (36%)	65 (40%)	0.571
Cholesterol levels				
Total Cholesterol (median [IQR]) (2 missing)	91.6 [74.0, 122.0]	78.0 [64.0, 97.3]	107.0 [87.0, 133.8]	< 0.001
HDL-C (median [IQR]), mg/dL	26.0 [15.0, 38.0]	18.0 [10.0, 30.9]	32.0 [20.0, 42.5]	< 0.001
LDL-C (median [IQR]) (7 missing), mg/dL	40.4 [26.0, 61.0]	29.0 [19.5, 42.0]	48.5 [34.1, 71.0]	< 0.001
Triglycerides (median [IQR]) (2 missing), mg/dL	114.0 [79.3, 153.0]	122.5 [84.3, 161.5]	108.5 [75.4, 145.8]	0.100
Severity, outcomes and clinical management				
SOFA Score (median [IQR])	7.0 [4.0, 10.0]	9.0 [7.0, 11.0]	5.0 [4.0, 7.5]	< 0.001
Vasopressor Use (n, %)	140 (49%)	84 (67%)	56 (34%)	< 0.001
Mechanical Ventilation (n, %)	104 (36%)	51 (41%)	53 (33%)	0.147
ICU LOS (median [IQR]) (3 missing)	4.0 [1.0, 7.0]	4.0 [2.0, 8.3]	3.0 [0.0, 6.0]	0.001
28-Day Mortality (n, %) (1 missing)	64 (22%)	38 (30%)	26 (16%)	0.004
Outcome (n, %)				
Chronic Critical Illness	51 (18%)	25 (20%)	26 (16%)	0.019
Early Death	35 (12%)	22 (18%)	13 (8%)	
Rapid Recovery	202 (70%)	78 (62%)	124 (76%)	

Comparison of clinical features including demographics, cholesterol levels, disease severity, and outcomes by HYPO vs. NORMO phenotypes. Rapid recovery was defined as clinical improvement and hospital discharge within 14 days, early death as in-hospital death within 14 days, and chronic critical illness (CCI) as intensive care unit stay of at least 14 days with organ dysfunction. HYPO = Hypolipoprotein; NORMO = Normolipoprotein; COPD = Chronic Obstructive Pulmonary Disease; ESRD = End Stage Renal Disease; HIV = Human Immunodeficiency Virus; IQR = Interquartile Range; HDL = High Density Lipoprotein; LDL = low density lipoprotein; SOFA = Sequential Organ Failure Assessment; ICU LOS = Intensive Care Unit Length of Stay

HYPO patients were clinically discernible from NORMO patients in several ways. A Seaborn clustermap provides a visual representation of the 15 defining features of HYPO and NORMO subphenotypes (Supplemental Fig. 1). The 15 features included triage temperature, triage systolic blood pressure, total cholesterol, LDL-C, HDL-C, paraoxonase-1 (PON1) activity, apolipoprotein (ApoA-I) levels, coagulation SOFA, intercellular adhesion molecule-1 (ICAM-1) level, hepatic SOFA, renal SOFA, total SOFA, cardio SOFA, neuro SOFA, and respiratory SOFA. HYPO patients exhibited lower total cholesterol (78, IQR 64–97.3 vs. 107, IQR 87–133.8, *p* < 0.001), HDL-C (18, IQR 10–30.9 vs. 32, IQR 20–42.5, *p* < 0.001), and LDL-C (29, IQR 19.5–42 vs. 48.5, IQR 34.1–71, *p* < 0.001) levels (mg/dL, Table 1) compared to NORMO. HYPO patients also had higher median SOFA scores (9, IQR 7–11 vs 5, IQR 4–7.5, *p* < 0.001), increased vasopressor use (67% vs 34%, *p* < 0.001), longer ICU lengths of stay (4 vs 3 days, *p* = 0.001), and higher 28-day mortality (30% vs 16%, *p* = 0.004) (Table 1) compared to NORMO. There was no significant differences in statin use vs. non-use in HYPO vs. NORMO patients, and there were no associated differences in SOFA score or mortality (Supplemental Table 2).

**Fig. 1:**
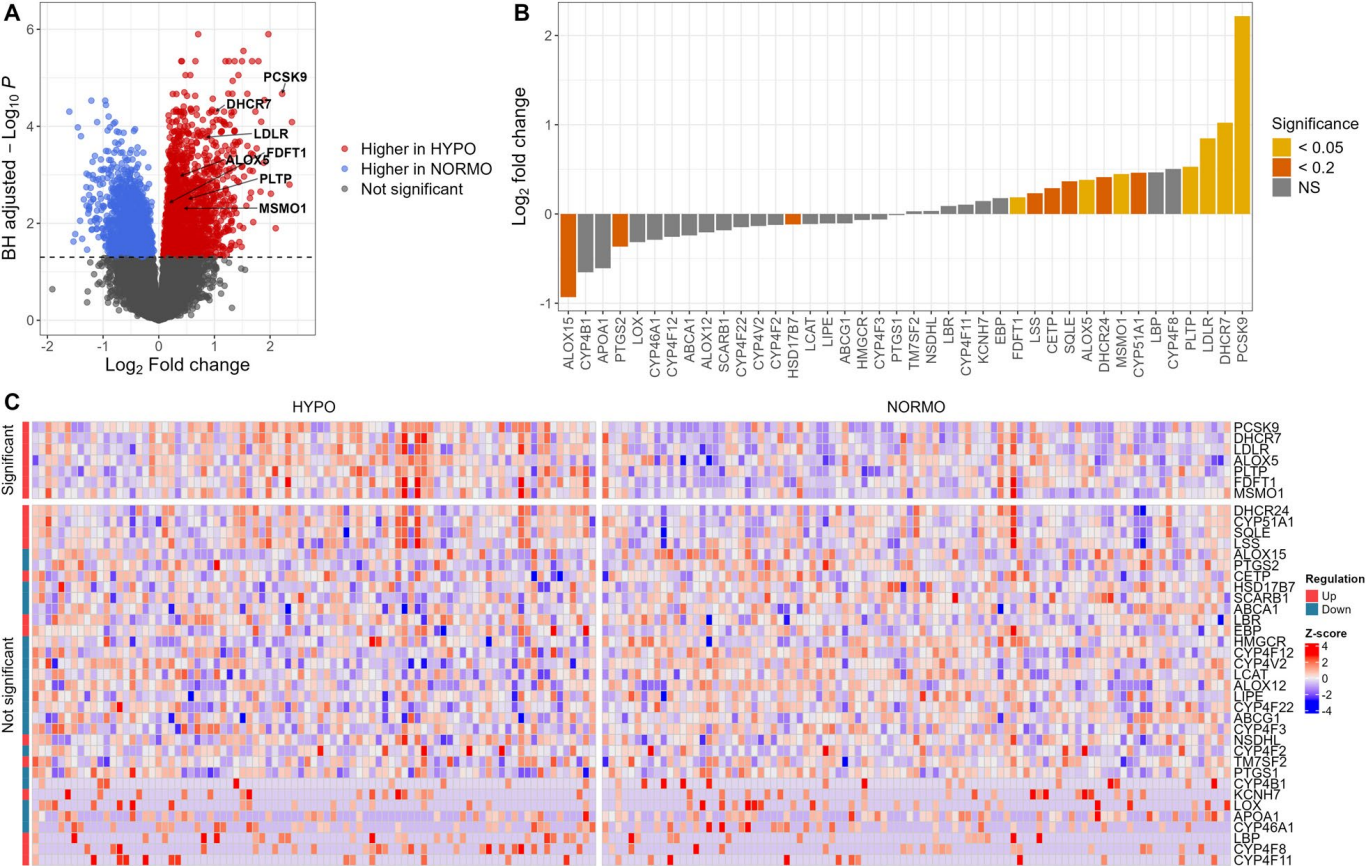
RNA-seq analysis comparing HYPO vs. NORMO. **A** Volcano plot displaying results from the differential expression analysis of 26,878 genes between HYPO and NORMO. Each dot represents a gene, with colors indicating significance, using a Benjamini-Hochberg-adjusted *P* value cutoff of less than 0.05 (dashed line). The x-axis denotes the log2 fold change for HYPO vs. NORMO, and the y-axis shows the Benjamini-Hochberg-adjusted -log10 *P* value. **B** Bar plot showing the log2 fold change of 40 lipid genes for HYPO vs. NORMO. Seven non-expressed lipid genes have been excluded from the analysis. Colors represent the significance of these genes, determined by the Benjamini-Hochberg-adjusted *P* value, adjusted for 26,878 comparisons. **C** Heatmap showing the expression of 40 lipid genes. Seven non-expressed lipid genes have been excluded from the analysis. The color scale corresponds to z-scored, log2-transformed gene expression values for each sample. Significance was determined by the Benjamini-Hochberg-adjusted *P* value, with a cutoff of 0.05, adjusted for 26,878 comparisons

**Table 2:** Differentially expressed lipid metabolism genes

Lipid gene	Total cohort mean (n = 184)	Log2 fold change^ (Standard error)	*p*-value	Adjusted *p*-value
*PCSK9*	68.231	2.217 (0.396)	< 0.001***	< 0.001*
*DHCR7*	253.077	1.023 (0.192)	< 0.001***	< 0.001*
*LDLR*	676.876	0.848 (0.174)	< 0.001**	< 0.001
*ALOX5*	23,052.467	0.382 (0.089)	< 0.001*	0.001
*PLTP*	35.984	0.529 (0.137)	< 0.001	0.003
*FDFT1*	966.266	0.186 (0.049)	< 0.001	0.004
*MSMO1*	166.634	0.448 (0.122)	< 0.001	0.005

Differentially expressed lipid metabolism genes in HYPO vs. NORMO patients. Seven out of the 47 a priori selected genes were differentially upregulated in HYPO compared to NORMO patients

*PCSK9* Proprotein Convertase Subtilisin/Kexin Type 9; *DHCR7* 7-Dehydrocholesterol Reductase; *LDLR* Low-Density Lipoprotein Receptor; *ALOX5* Arachidonate 5-Lipoxygenase; *PLTP* Phospholipid Transfer Protein; *FDFT1* Farnesyl-Diphosphate Farnesyltransferase 1; *MSMO1* Methylsterol Monooxygenase 1

^ Fold change was calculated using mean of HYPO (n = 87)/mean of NORMO (n = 97)

Significance codes: *p < 0.0001, **p < 0.00001, ***p < 0.000001

RNAseq was conducted on samples from 184 patients. Of these, 87 (47%) were classified as HYPO and 97 (53%) were NORMO. Seven lipid metabolism genes were upregulated in HYPO (Table 2) compared to NORMO patients. These genes were *Proprotein Convertase Subtilisin/Kexin Type 9 (PCSK9), 7-Dehydrocholesterol Reductase (DHCR7), Low-Density Lipoprotein Receptor (LDLR), Arachidonate 5-Lipoxygenase (ALOX5), Plasma Phospholipid Transfer Protein (PLTP), Farnesyl-Diphosphate Farnesyltransferase 1 (FDFT1), and Methylsterol Monooxygenase 1 (MSMO1)*. The volcano plot (Fig. 1A) illustrates upregulated genes, with lipid genes annotated, in HYPO vs. NORMO patients. Figure 1B demonstrates the relative up vs. downregulation of each gene contributing to the HYPO vs. NORMO subphenotypes. Figure 1C displays a Seaborn clustermap of gene expression by subphenotype. Of the 47 a priori lipid-related genes of interest (Supplemental Table 3), 37 were cataloged in the KEGG database. The primary pathways associated with these significant genes include steroid biosynthesis, cholesterol metabolism, and arachidonic acid metabolism as detailed in the Supplemental Data File. Three additional KEGG pathways—ovarian steroidogenesis, serotonergic synapse, and efferocytosis—were also enriched in our analysis when using significance criterion for significant genes to an adjusted *p* = 0.2. Supplemental Fig. 2 displays boxplots of the upregulated genes in HYPO vs. NORMO patients as well as the mean contribution of each gene to the HYPO subphenotype via the Gini coefficient (GC).

**Fig. 2:**
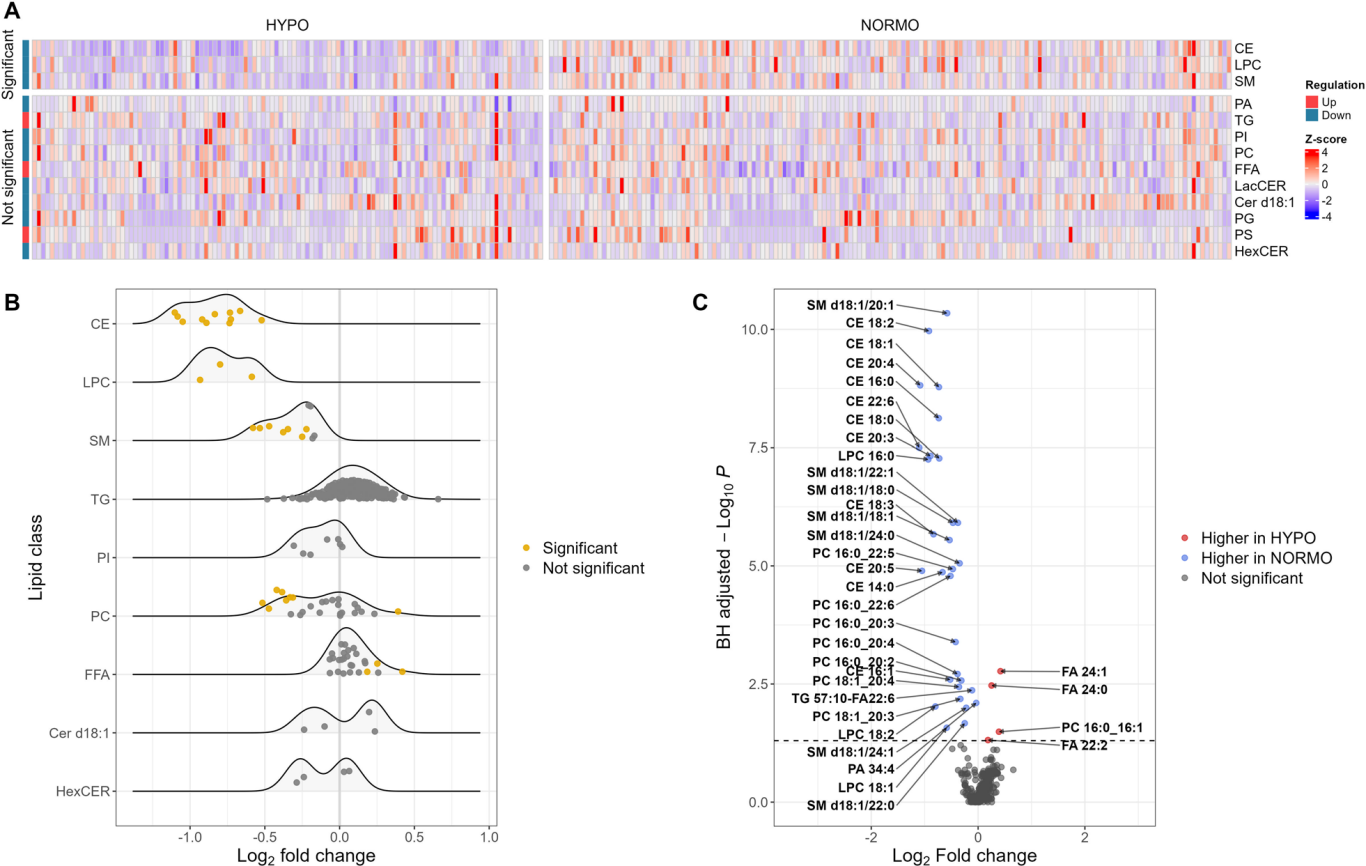
Lipidomics analysis comparing HYPO vs. NORMO. **A** Heatmap showing the abundance of 13 lipid classes analyzed via shotgun lipidomics. The color scale corresponds to z-scored concentration values for each sample. Significance was determined by the Benjamini-Hochberg-adjusted *P* value, with a cutoff of 0.05, adjusted for 13 lipid class comparisons. **B** Ridgeline plot comparing lipid species between HYPO and NORMO. Each dot represents an individual lipid species within its corresponding lipid class (y-axis). The color of the dot indicates whether the lipid is significantly altered, with a cutoff of 0.05 for the Benjamini-Hochberg-adjusted *P* value, adjusted for all 355 lipid species comparisons. The x-axis represents the log2 fold change for HYPO vs. NORMO. Four lipid classes (PA, LacCER, PG, and PS) are hidden due to having fewer than 3 individual lipid species within the class. **C** Volcano plot displaying the differential abundance of 355 lipids between HYPO and NORMO. Each dot represents a lipid species, with colors indicating significance using a Benjamini-Hochberg-adjusted *P* value cutoff of less than 0.05. The x-axis denotes the log2 fold change for HYPO vs. NORMO, and the y-axis shows the Benjamini-Hochberg-adjusted -log10 *P* value

Shotgun lipidomics analysis was performed on 271 patients, with 116 (43%) classified as HYPO and 155 (57%) classified as NORMO. HYPO patients showed significantly lower levels of specific classes of lipids including cholesterol esters (CE, adjusted *p* < 0.001), sphingomyelins (SM, adjusted *p* < 0.001), and lysophosphatidylcholines (LPC, adjusted *p* < 0.001) (Supplemental Table 4). Individual lipids that were significantly different between classes are displayed in Supplemental Table 5, most of which had lower levels in HYPO vs. NORMO patients. Figure 2A shows the Seaborn clustermap of lipid classes by HYPO vs. NORMO subphenotype. Figure 2B shows the differences in lipid classes between HYPO and NORMO subphenotypes, while Fig. 2C demonstrates the log_2_ fold change in individual lipid moieties between HYPO vs. NORMO patients. Our signaling lipid panel was conducted on 257 patients, with 111 (43%) classified as HYPO and 146 (57%) classified as NORMO. However, the results were not statistically significant between the two cohorts for any signaling lipids (Supplemental Table 6).

**Fig. 3:**
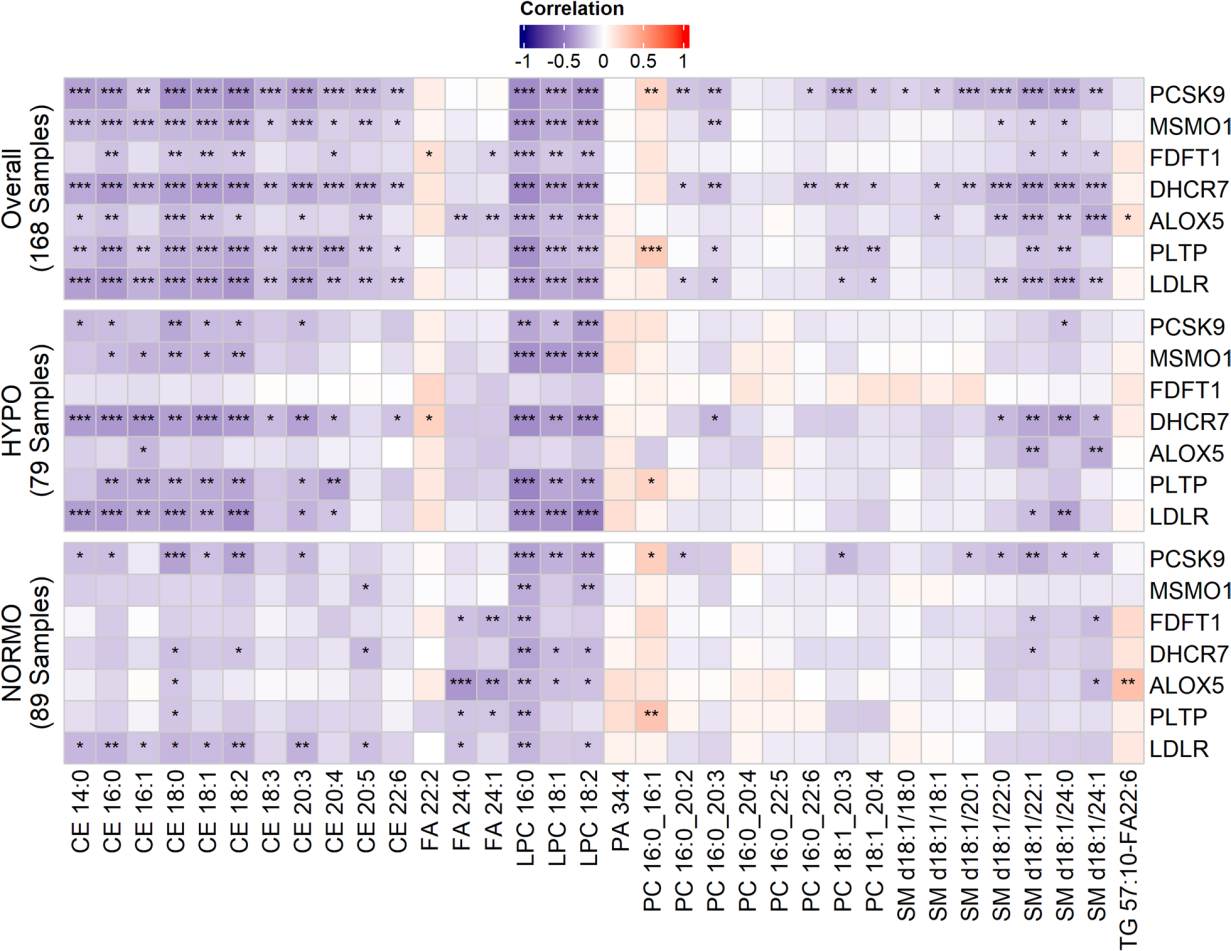
Correlation analysis between individual lipids and HYPO vs. NORMO subphenotypes by expression of the 7 significant genes. The correlation matrix displays significant correlations between genes in overall analysis (all patients, N = 168), HYPO patients only (N = 79), and NORMO patients only (N = 89), for patients with RNAseq data. Significant correlations are indicated as follows: ***p < 0.001, **p < 0.01, *p < 0.05). Differences in the correlations between specific upregulated genes and individual cholesterol esters (CE), lysophosphatidylcholines (LPC), phosphatidylcholines (PC), and sphingomyelins (SM) are observable between HYPO and NORMO patients, with the most significant differences correlated with upregulation of *PCSK9, MSMO1, DHCR7, PLTP,* and *LDLR* in HYPO patients

To better understand the association between upregulated genes by subphenotype, and specific lipids in the shotgun lipidomics experiment, we visually presented these data using a correlation matrix (Fig. 3). Here we can see that the influence of gene expression differences between HYPO and NORMO subphenotypes on individual lipid levels becomes apparent. Specifically, *DHCR7* and *LDLR* upregulation in the HYPO cohort was strongly correlated with reduced levels of CE 14:0, 16:0, 16:1, CE 18:0, CE 18:1, CE 18:2, and CE 20:3, compared to the NORMO cohort. Upregulation of *PCSK9, MSMO1, DHCR7, PLTP,* and *LDLR* in HYPO patients was also more strongly correlated with upregulation of LPC’s 18:0, 18:1, and 18:2 compared to NORMO patients overall. *DHCR7* upregulation in HYPO was also most strongly correlated with low SM levels, specifically d18:1/22:1 and d18:1/24:0. Interestingly, *PCSK9* expression in NORMO patients was more strongly associated with low SM levels than in HYPO patients and was also significantly associated with low LPCs.

We compared the long-term survival of HYPO vs. NORMO patients. Using the log-rank test with 95% confidence intervals and comparing survival of patients out to 1 year, 6-month survival was 63.7% (95% CI 54.5–74.5) for HYPO patients, and 78.6% (95% CI 71–87) for NORMO patients (*p* = 0.0085). At one year, survival was 56.2% (95% CI 46.1–68.6) for HYPO patients and 73% (95% CI 64.4–82.7) for NORMO patients (*p* = 0.0067, Supplemental Fig. 3).

### External dataset comparisons

Differential expression analysis was conducted on two external datasets [32, 33]. The study by Scicluna et al. included sepsis patients who were admitted to two ICUs in the Netherlands, and described four phenotypes based on gene expression patterns, designated MARS 1, 2, 3, and 4. Patients with MARS 1 or 2 phenotypes had the highest burden of organ failure, shock, and the highest mortality compared to patients with MARS 3 or 4 phenotypes. We performed differential expression analysis on 479 MARS sepsis patients focused on our 47 a priori lipid metabolism genes. Comparing 28-day non-survivors to survivors, non-survivors had upregulation of two lipid metabolism genes *TM7SF2* and *APOA1* at the adjusted *p* < 0.05 level, and five genes *FDFT1, LDLR, MSMO1, EBP,* and *LOX* at adjusted *p* < 0.2. Three of these genes, *FDFT1, LDLR,* and *MSMO1*, were the same as those identified in the HYPO subphenotype. When comparing the more severe MARS 1 and 2 patients to MARS 3 and 4 patients, MARS 1 and 2 patients had upregulation of six of the seven genes as our HYPO subphenotype including *ALOX5, FDFT1, LDLR, MSMO1, DHCR7,* and *PCSK9* at adjusted *p* < 0.05. We subsequently applied the random forest models to predict HYPO and NORMO groups within the MARS data. The more critically ill MARS 1 or 2 group contained more HYPO (52%, 159/308) than NORMO patients, while the less critically ill MARS 3 or 4 group contained fewer HYPO (20%, 34/171) than NORMO patients (*p* < 0.001). However, the proportion of predicted HYPO among 28-day non-survivors (46%, 53/114) vs. survivors (38%, 140/365) was not significantly different (*p* = 0.15).

**Fig. 4:**
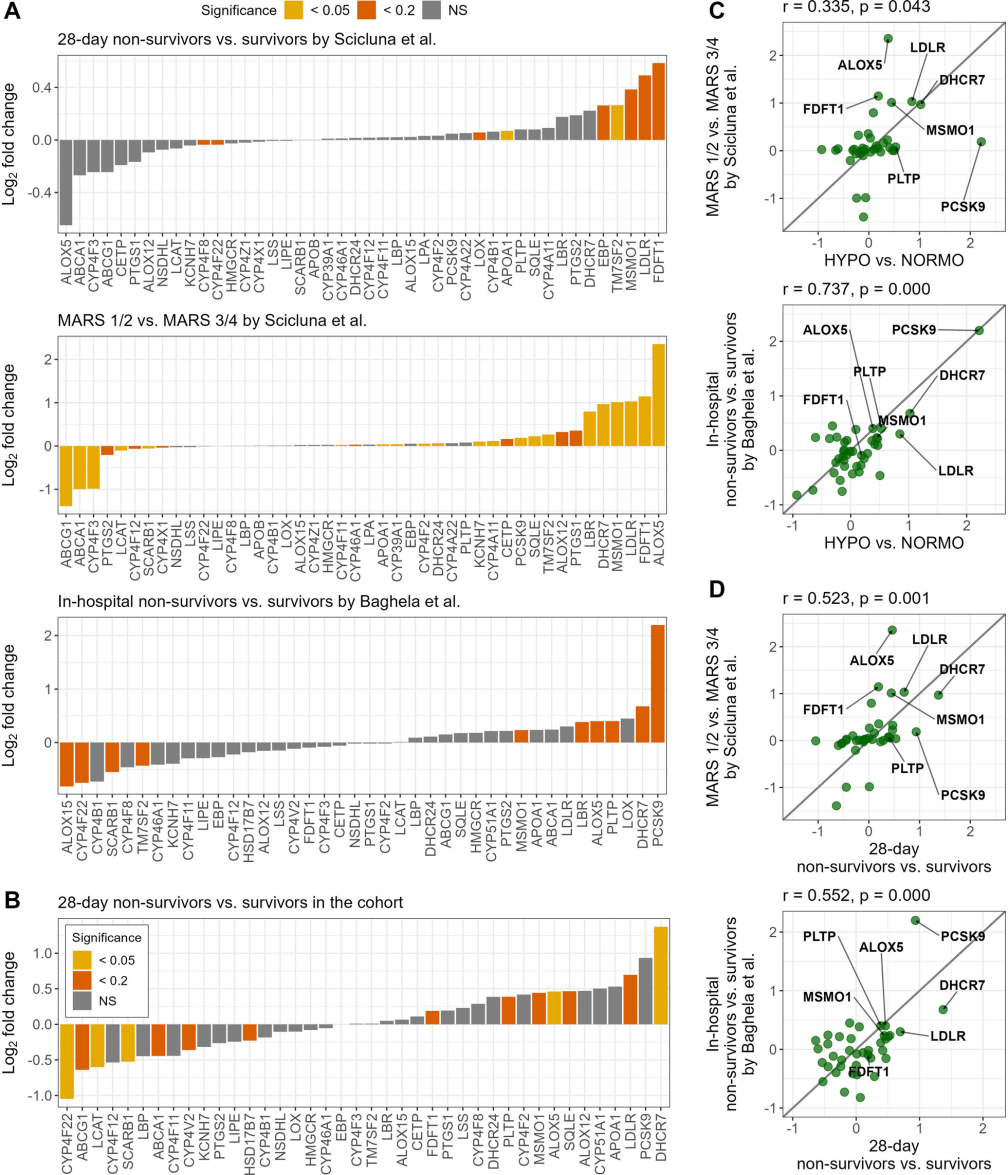
Comparison of gene expression patterns in external datasets. **A** Bar plot showing the log2 fold changes of lipid genes for 28-day non-survivors vs. survivors, MARS 1/2 vs. MARS 3/4 (Scicluna et al.), and in-hospital non-survivors vs. survivors (Baghela et al.). Non-expressed lipid genes have been excluded from the corresponding analysis. Colors represent the significance of these genes, determined by the Benjamini-Hochberg-adjusted *P* value. **B** Bar plot showing the log2 fold changes of 40 lipid genes for 28-day non-survivors vs. survivors in our study. Seven non-expressed lipid genes have been excluded from the analysis. Colors represent the significance of these genes, determined by the Benjamini-Hochberg-adjusted *P* value. **C** Correlation between log2 fold changes from validation sets compared to HYPO vs. NORMO in our study. The x-axis denotes the log2 fold change for HYPO vs. NORMO in our study, and the y-axis shows the log2 fold changes for MARS 1/2 vs. MARS 3/4 (Scicluna et al.) and in-hospital non-survivors vs. survivors (Baghela et al.), separately. **D** Correlation between log2 fold changes from validation sets compared to 28-day non-survivors vs. survivors in our study. The x-axis denotes the log2 fold change for 28-day non-survivors vs. survivors in our study, and the y-axis shows the log2 fold changes for MARS 1/2 vs. MARS 3/4 (Scicluna et al.) and in-hospital non-survivors vs. survivors (Baghela et al.), separately

The second study, by Baghela et al., included 345 sepsis patients. We compared patients by in-hospital mortality and found that five of our seven genes, *PCSK9, DHCR7, ALOX5, PLTP,* and *MSMO1* were upregulated (adjusted *p* < 0.2). *ALOX15* was the most downregulated lipid metabolism gene in non-survivors in this cohort, as it was in HYPO patients. These comparisons are displayed in Fig. 4**.** Finally, we performed a correlation analysis and found that gene expression patterns of HYPO vs. NORMO correlated with MARS 1/2 vs. MARS 3/4 expression patterns (r = 0.335 and *p* = 0.043), and in-hospital mortality in the study by Baghela et al. (r = 0.737, *p* < 0.001). Similarly, when comparing by 28-day mortality of patients in our study, these were still correlated with MARS 1/2 vs. 3/4 expression patterns (r = 0.523, *p* = 0.001), and in-hospital mortality in the study by Baghela et al. (r = 0.552, *p* < 0.001). Finally, we applied the random forest models to predict the HYPO vs. NORMO patients within the study and examined the difference in the predicted proportion of HYPO patients by in-hospital mortality. We found that the difference was not significant (*p* = 0.55), with 35% (18/52) of non-survivors classified as HYPO and 29% (86/293) of survivors classified as HYPO, though the overall number of non-survivors was lower than the Scicluna et al. study.

## Discussion

Our study findings reveal intriguing insights into the molecular pathways of lipid dysregulation in sepsis, and specifically to our HYPO and NORMO sepsis subphenotypes. Seven genes involved in cholesterol biosynthesis, endotoxin clearance, steroid production, and lipid metabolism were upregulated in HYPO patients. Lipidomics analysis showed significantly lower levels of CEs, SMs, and LPCs. The dysregulated lipid and lipoprotein metabolic profile observed in HYPO patients suggests a sepsis subphenotype where cholesterol is being eliminated from circulation to facilitate bacterial toxin clearance, and/or is being utilized as substrate for steroid production, cell membranes, and other physiological needs to defend against sepsis. In two external datasets, compared by mortality and sepsis severity, we identified six of the same upregulated genes- *PCSK9, DHCR7, ALOX5, PLTP, LDLR,* and *MSMO1*.

This study is the first to conduct comprehensive analyses integrating large-scale RNA sequencing (RNAseq) and lipidomics data into one study from a diverse sepsis cohort using a lipoprotein-based phenotyping approach. The advantage of our approach is the ability to understand and interpret changes in the plasma lipidome in sepsis, in the context of genetic upregulation of specific genes. Here, we identified seven upregulated lipid metabolism genes in circulating leukocytes in HYPO sepsis patients, namely, *PCSK9, DHCR7, LDLR, ALOX5, PLTP, FDFT1,* and *MSMO1*. Three (*PCSK9, LDLR, and PLTP*) are involved in endotoxin clearance [37–[39]. *DHCR7* catalyzes a critical step in cholesterol biosynthesis [40]. *FDFT1* is the first enzyme in cholesterol biosynthesis [41]. *MSMO1* catalyzes a three-step mono-oxygenation step, which can be metabolized to cholesterol [42]. Lastly, *ALOX5* generates 5-HETE, leading to leukotriene production [43]. It is unknown whether targeting these genes could lead to personalized medicine or if genetic upregulation merely indicates membership into a subphenotype of patients with more dysregulated lipid metabolism. Our correlation analysis also provided a deeper understanding of the relationship between the upregulation of specific lipid metabolism genes and individual lipid levels. In HYPO patients, *DHCR7* expression strongly correlated with reductions in CE, LPC, and SM, while *PCSK9, MSMO1, DHCR7, PLTP,* and *LDLR* upregulation were correlated with low LPC. *DHCR7, ALOX5*, and *LDLR* correlated most strongly with reductions in SM. Interestingly, *PCSK9* in NORMO patients was also associated with low SMs and LPCs and *ALOX5* was associated with elevated triglyceride TG57:10-FA22:6.

Targeting lipid metabolism genes as novel therapy for sepsis may have potential. Engoren et al. unveiled an association between the *PCKS9* variant and a nearly two fold increase in the likelihood of developing sepsis, as defined by both the Sepsis-2 and Sepsis-3 criteria [11]. Presently, clinical trials are underway of two PCKS9 inhibitors, alirocumab (NCT03634293) and evolocumab (NCT03869073), aimed at evaluating their efficacy in reducing mortality due to sepsis. Reyes et al. studied immune dysregulation in bacterial sepsis by clustering gene expression profiles [12]. They found a unique expanded CD14 + monocyte state in septic patients, which could distinguish them from controls using public transcriptomic data. *ALOX5*, a marker gene highly expressed in this monocyte group, was also identified in our study, suggesting its potential for classifying sepsis patients from those with sterile inflammation. Furthermore, Zhang et al. identified *ALOX5* as one of the 15 mRNAs likely to demonstrate strong diagnostic utility for pediatric sepsis [13]. The findings of these studies, combined with the results of this study, provide compelling evidence of the association between upregulation of specific lipid genes in septic patients and a subgroup of patients with dysregulated lipid metabolism at risk of poor outcomes.

Our findings across two external datasets with 824 combined patients, demonstrate the importance of the seven identified lipid genes. Several of the genes expressed in our subphenotype were upregulated in both cohorts for the mortality comparison, though more strongly in the study by Baghela et al. *FDFT1, LDLR,* and *MSMO1* were in common for both mortality comparisons, indicating common upregulation of cholesterol biosynthesis and clearance pathways in critically ill sepsis patients with increased mortality. When comparing our 28-day mortality expression patterns to that of Scicluna et al. and Baghela et al., we found that *DHCR7* and *ALOX5* (adjusted *p* < 0.05), as well as *LDLR, SQLE, MSMO1, PLTP,* and *FDFT1* were also upregulated (adjusted *p* < 0.2). However, most striking was the uniform upregulation of five of our seven genes in the MARS 1 and 2 vs. 3 and 4 patients, which showed six of the same genes upregulated as seen in HYPO vs. NORMO patients at the adjusted *p* < 0.05 level (*ALOX5, FDFT1, LDLR, MSMO1, DHCR7, PCSK9*). This may indicate common lipid dysregulation pathways between these subphenotypes and among sepsis patients with greater disease severity. The downregulation of *ALOX15*, both in our HYPO vs. NORMO comparison, and in the in-hospital mortality comparison for the Baghela et al. study, may indicate important downregulation of pro-resolving anti-inflammatory lipids and metabolites of eicosanoids, which may potentiate the dysregulated inflammation of sepsis. [44]

Our lipidomics analysis revealed findings consistent with Chouchane et al. [10] They observed reduced cholesterol esters and lysophospholipids in sepsis patients and noted that cholesterol ester recovery at 4 days was associated with reduced 30-day mortality. Our HYPO subphenotype similarly demonstrated reduced CE levels compared to NORMO in the first 24 h. Cholesterol esters are a storage form of free cholesterol in plasma [45]. Taken together with the upregulation of cholesterol biosynthesis genes (*DHCR7*), and genes that speed the elimination of cholesterol from circulation (*PCSK9, LDLR*), reduced CE levels likely indicate cholesterol utilization *and* elimination from circulation. Specifically, free cholesterol is likely being utilized as a substrate for steroid production and cell membrane production and to meet other physiologic needs, while also being eliminated via hepatic SR-BI receptors bound to HDL and LDL for bacterial toxin clearance [45]. The reduced levels of lysophosphatidylcholines (LPCs) and sphingomyelins (SMs) in HYPO sepsis may also indicate a decrease in innate immunity [46–48]. LPCs, a major component of oxidized LDL, are known to be reduced in sepsis non-survivors [49]. They play a protective role against lethal sepsis by stimulating neutrophils to eliminate invading pathogens through an H_2_O_2_-dependent mechanism [46]. SMs are a major plasma and cell membrane component [50]. Certain bacterial toxins trigger the conversion of SMs to ceramides by sphingomyelinase, leading to localization of lysosomes and release of cathepsin B and D from lysosomes in the cytoplasm, leading to the formation of 1L-1β and TNF-α [48]. SMs conversion to ceramides also stimulates neutrophil extracellular trap (NET) formation, which can be augmented by drugs such as tamoxifen (FDA-approved breast cancer drug) [51].

Our study highlights critical lipid dysregulation pathways in sepsis with significant potential for precision medicine. In the HYPO sepsis subphenotype, seven upregulated genes involved in cholesterol biosynthesis, endotoxin clearance, and lipid metabolism were identified, along with decreased levels of cholesterol esters (CEs), sphingomyelins (SMs), and lysophosphatidylcholines (LPCs). This dysregulation suggests that cholesterol is redirected for essential immune functions and bacterial toxin clearance. Comparison across two external datasets supports the clinical relevance of these genes, particularly in relation to mortality risk.

By integrating lipid-related biomarkers into diagnostic tools, we can facilitate early detection and risk stratification for high-risk patients. Identifying distinct sepsis subphenotypes with varying morbidity and mortality levels enables stratified care tailored to specific lipid profiles. Moreover, ongoing advancements in health informatics make it feasible to integrate multiomic data into electronic health record (EHR) systems, allowing for the development of risk stratification algorithms that classify patients by subphenotype and guide personalized care. Targeting genes like *PCSK9* and DHCR7 could optimize treatments and improve outcomes, paving the way for a precision approach to sepsis management.

This study's limitations include its single-site nature, potentially limiting generalizability. However, the sizable and diverse cohort, balanced by age, sex, and race, and genetic validation in two independent cohorts strengthens the findings. The observational design limits causal inference, identifying only associations between genes or lipids and each subphenotype. While the study provides valuable insights into transcriptomic and lipidomic dysregulation in HYPO and NORMO sepsis patients, further research is needed to establish causal relationships. An additional limitation is the absence of protein expression and enzymatic activity measurements for the differentially expressed lipid metabolism genes. While the study suggests that upregulation of these genes may lead to reduced levels of specific lipids, the activity of the expressed enzymes was not assessed. Measuring protein or enzymatic activity could be a next step toward establishing causality.

## Conclusion

In this study, HYPO sepsis patients had upregulation of seven lipid metabolism genes for cholesterol biosynthesis and clearance, and regulation of inflammation, of which six were identified in validation studies by mortality and phenotype comparisons. Five genes in HYPO sepsis patients were most strongly correlated with low CE, LPC, and SMs that mediate cholesterol storage and innate immunity. HYPO patients were clinically discernible by higher disease severity and lower one-year survival. Future studies will investigate the potential of these genes and lipids to serve as targets for sepsis precision medicine.

## Supplementary Information

Supplementary file 1. Supplemental data file

Supplementary file 2. Supplementary Fig. 1. Seaborn clustermap of HYPO and NORMO subphenotypes and the 15 defining features. Seaborn clustermap provides a visual representation of the 15 defining features of HYPO and NORMO phenotypes. Each bar represents a patient. The yellow bars indicates 28-day mortality, while green bars indicate 28-day survival.

Supplementary file 3. Supplementary Fig. 2. Analysis of 7 significant lipid genes as markers for distinguishing HYPO from NORMO. (A) Boxplot displaying the expression levels of the 7 significant lipid genes in HYPO and NORMO groups. The y-axis shows the log2-transformed normalized gene expression levels for each group. (B) Gini importance scores of the 7 significant lipid genes for predicting HYPO or NORMO classification using a random forest model.

Supplementary file 4. Supplementary Fig. 3. 1-year survival analysis comparing HYPO vs. NORMO. Survival curve displaying the 1-year survival of HYPO vs. NORMO patients over time. There were 208 patients included with recorded survival data.

Supplementary file 5. Supplemental flow diagram.

Supplementary file 6. Supplemental tables and methods section.

## Data Availability

Data is available upon reasonable request by contacting fguirgis@ufl.edu and will be available in dbGaP after article publication.
