# Supplementary figures and images for: Multiomic molecular patterns of lipid dysregulation in a subphenotype of sepsis with higher shock incidence and mortality
Source: Crit Care. 2024 Dec 24;28:431. doi: 10.1186/s13054-024-05216-3 (PMC11667828; doi:10.1186/s13054-024-05216-3)

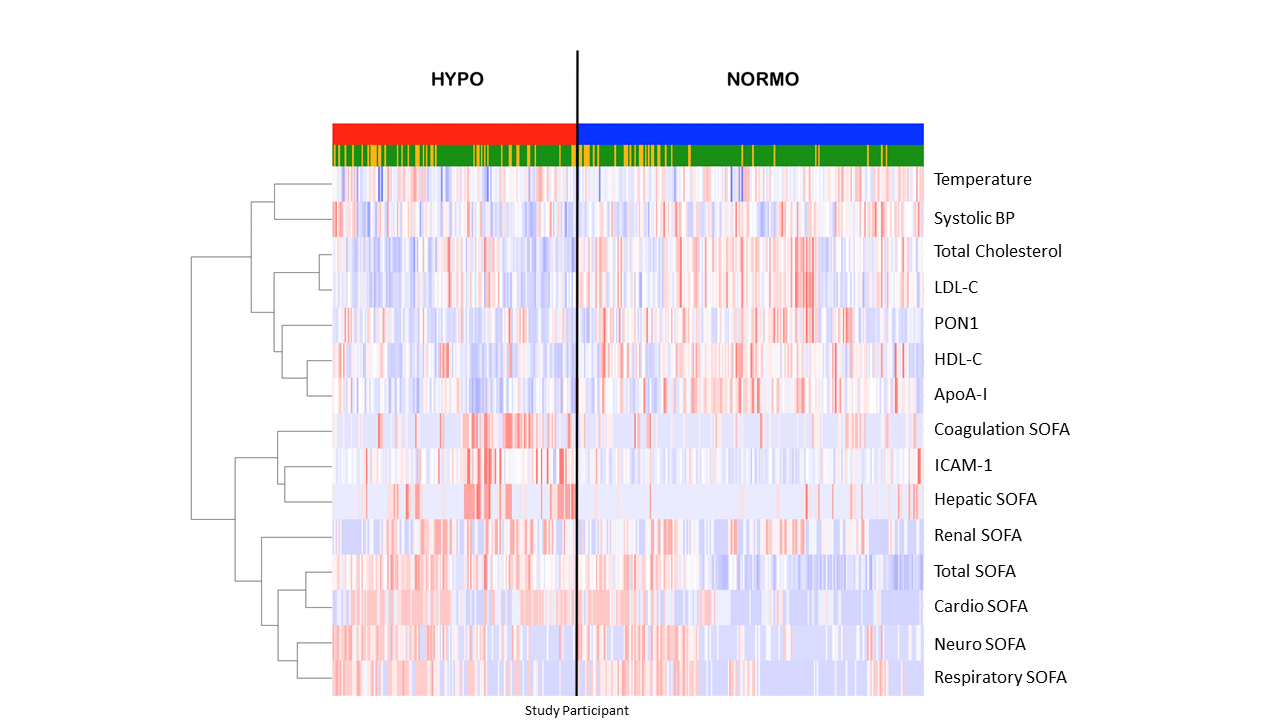

Supplement: Supplementary file 2 — Supplementary Fig. 1. Seaborn clustermap of HYPO and NORMO subphenotypes and the 15 defining features. Seaborn clustermap provides a visual representation of the 15 defining features of HYPO and NORMO phenotypes. Each bar represents a patient. The yellow bars indicates 28-day mortality, while green bars indicate 28-day survival. [file 13054_2024_5216_MOESM2_ESM.tif]

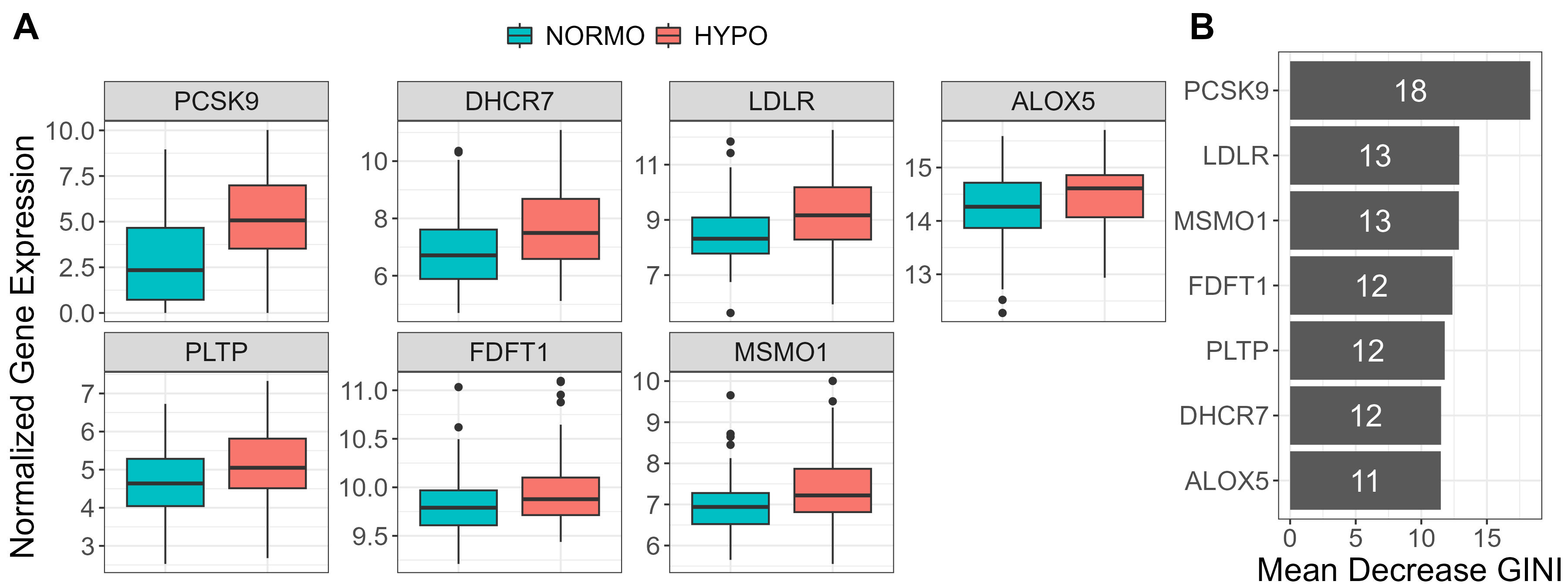

Supplement: Supplementary file 3 — Supplementary Fig. 2. Analysis of 7 significant lipid genes as markers for distinguishing HYPO from NORMO. (A) Boxplot displaying the expression levels of the 7 significant lipid genes in HYPO and NORMO groups. The y-axis shows the log2-transformed normalized gene expression levels for each group. (B) Gini importance scores of the 7 significant lipid genes for predicting HYPO or NORMO classification using a random forest model. [file 13054_2024_5216_MOESM3_ESM.jpg]

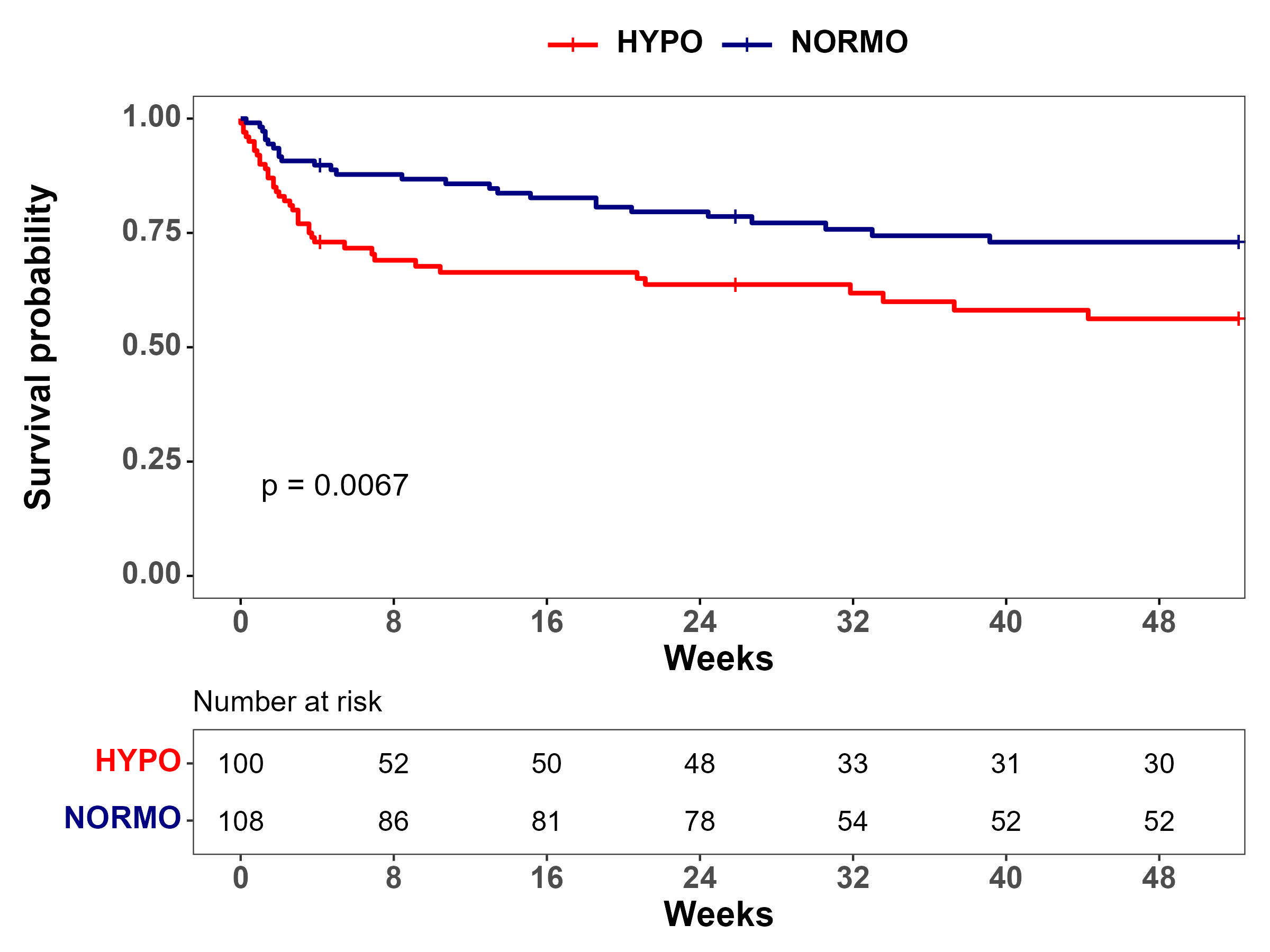

Supplement: Supplementary file 4 — Supplementary Fig. 3. 1-year survival analysis comparing HYPO vs. NORMO. Survival curve displaying the 1-year survival of HYPO vs. NORMO patients over time. There were 208 patients included with recorded survival data. [file 13054_2024_5216_MOESM4_ESM.jpg]

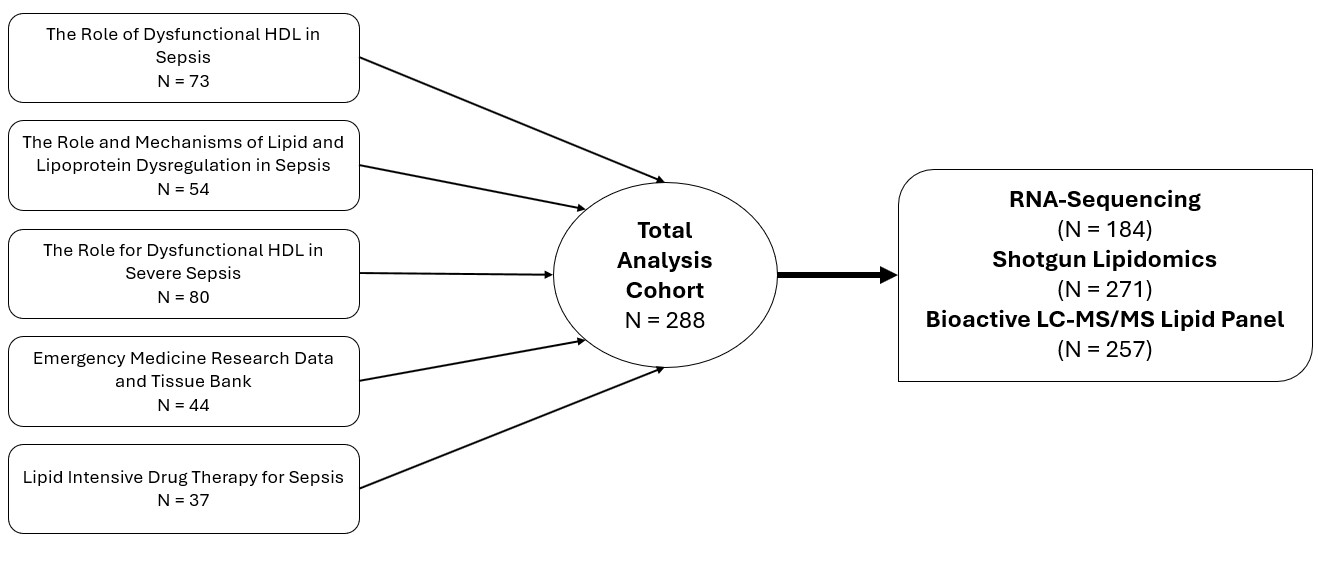

Supplement: Supplementary file 5 — Supplemental flow diagram. [file 13054_2024_5216_MOESM5_ESM.jpg]
