# Supplementary material for: Multiomic molecular patterns of lipid dysregulation in a subphenotype of sepsis with higher shock incidence and mortality
Source: Crit Care. 2024 Dec 24;28:431. doi: 10.1186/s13054-024-05216-3 (PMC11667828; doi:10.1186/s13054-024-05216-3)
Supplement: Supplementary file 6 — Supplemental tables and methods section. [file 13054_2024_5216_MOESM6_ESM.docx]

**Supplemental Tables and Methods**

Table of Content

| Content | Page Number |
| --- | --- |
| Supplemental Table 1: Source of Infection  Supplemental Table 2. Statin Use by Subphenotype  Supplemental Table 3. Lipid Genes of Interest  Supplemental Table 4: Differences in Circulating Lipid Classes  Supplemental Table 5: Shotgun Lipidomics Individual Lipids  Supplemental Table 6. Lipid Panel Results | 2  3  4  5  6-7  8 |
| Shotgun Lipidomics Methods  External Data Set Preparation and Processing | 9-10  11 |
| References | 12 |
|  |  |
|  |  |

**Supplemental Table 1. Source of Infection**

| **Infection Source**  **(n, %)** | **Total Cohort**  **(n = 288)** | **HYPO Cohort**  **(n = 125)** | **NORMO Cohort**  **(n = 163)** |
| --- | --- | --- | --- |
| Blood | 9 (3%) | 4 (3%) | 5 (3%) |
| Bone / Osteomyelitis | 6 (2%) | 2 (2%) | 4 (2%) |
| CNS / Meningitis | 1 (0%) | 1 (1%) | 0 (0%) |
| Endocarditis | 9 (3%) | 5 (4%) | 4 (2%) |
| Intra-Abdominal | 26 (9%) | 16 (13%) | 10 (6%) |
| IV | 2 (1%) | 2 (2%) | 0 (0%) |
| Multiple | 9 (3%) | 4 (3%) | 5 (3%) |
| Necrotizing Soft Tissue | 2 (1%) | 0 (0%) | 2 (1%) |
| Other | 6 (2%) | 2 (2%) | 4 (2%) |
| Pulmonary | 90 (31%) | 31 (25%) | 59 (36%) |
| Skin / Soft Tissue | 28 (10%) | 11 (9%) | 17 (10%) |
| Surgical Site | 3 (1%) | 1 (1%) | 2 (1%) |
| Surgical Thoracic | 2 (1%) | 2 (2%) | 0 (0%) |
| Unknown | 6 (2%) | 2 (2%) | 4 (2%) |
| Urinary Tract | 89 (31%) | 42 (34%) | 47 (29%) |

**Supplemental Table 1.** The different sources of infection identified in patients with sepsis, the number and percentage of patients with each infection source in both cohorts, and the statistical significance. HYPO = Hypolipoprotein; NORMO = Normolipoprotein.

**Supplemental Table 2. Statin use compared by HYPO vs. NORMO subphenotypes for SOFA score and 28-day mortality.**

|  | HYPO Cohort  (n = 123)  (2 missing Statin Use) | | | NORMO Cohort  (n = 163) | | |
| --- | --- | --- | --- | --- | --- | --- |
|  | Statin Use  (n = 45) | No Statin Use (n = 78) | P-Value | Statin Use (n = 65) | No Statin Use (n = 98) | P-Value |
| SOFA Score (median [IQR]) | 9.0  [7.0, 11.0] | 9.0  [7.0, 11.0] | 0.804 | 5.0  [4.0, 8.0] | 5.0  [4.0, 7.0] | 0.622 |
| 28-Day Mortality  (n, %)  (1 missing) | 17 (38%) | 20 (26%) | 0.157 | 7 (11%) | 19 (19%)  (1 missing) | 0.134 |

**Supplemental Table 4.** Statin use vs. non-use presented by HYPO and NORMO subphenotypes and associations with SOFA score and 28-day mortality.

**Supplemental Table 3. Lipid Metabolism Genes of Interest**

| **Lipid Metabolism Genes** | | | |
| --- | --- | --- | --- |
| DHCR7 | CYP4Z1 | CYP51A1 | LDLR |
| KCNH7 | CYP4X1 | CYP46A1 | APOA1 |
| EBP | CYP4V2 | CYP39A1 | SCARB1 |
| HSD17B7 | CYP4F22 | ALOX15 | ABCG1 |
| NSDHL | CYP4F12 | ALOX12 | ABCA1 |
| MSMO1 | CYP4F11 | LOX | LIPE |
| TM7SF2 | CYP4F8 | PTGS1 | LPA |
| LBR | CYP4F3 | APOB | LBP |
| DHCR24 | CYP4F2 | HMGCR | CETP |
| LSS | CYP4B1 | PTGS2 | PLTP |
| SQLE | CYP4A22 | ALOX5 | LCAT |
| FDFT1 | CYP4A11 | PCSK9 |  |

**Supplemental Table 3.** 47 *a prior* lipid genes of interest selected for study in this investigation.

**Supplemental Table 4. Differences in Circulating Lipid Classes**

| **Lipid Class** | **Total Cohort**  **(n = 271)** | **HYPO Cohort**  **(n = 116)** | **NORMO Cohort**  **(n = 155)** | **Adjusted**  **P-value^** |
| --- | --- | --- | --- | --- |
| CE  (median [IQR]) | 1200.402  [771.271, 1611.616] | 810.460  [518.247, 1304.225] | 1424.16  [1080.028, 1833.432] | <0.001*** |
| LPC  (median [IQR]) | 25.322  [15.360, 42.773] | 18.097  [10.160, 32.000] | 32.349  [20.852, 47.361] | <0.001** |
| SM  (median [IQR]) | 248.993  [203.143, 307.481] | 220.823  [186.311, 278.423] | 271.356  [218.637, 316.372] | <0.001 |
| PA  (median [IQR]) | 2.739  [2.660, 2.849] | 2.730  [2.630, 2.853] | 2.744  [2.671, 2.842] | 0.368 |
| PC  (median [IQR]) | 1223.862  [986.740, 1474.820] | 1139.374  [941.996, 1405.988] | 1269.612  [1035.440, 1494.978] | 0.390 |
| PI  (median [IQR]) | 32.830  [25.065, 45.173] | 29.854  [22.846, 41.444] | 35.070  [26.949, 46.972] | 0.390 |
| TG  (median [IQR]) | 835.939  [579.697, 1302.309] | 890.602  [631.838, 1428.644] | 809.843  [561.457, 1194.530] | 0.390 |
| FFA  (median [IQR]) | 668.903  [562.960, 759.210] | 677.999  [576.431, 767.898] | 664.822  [561.878, 755.332] | 0.532 |
| LacCER  (median [IQR]) | 2.959  [2.268, 3.665] | 2.773  [2.159, 3.747] | 3.023  [2.420, 3.622] | 0.704 |
| Cer d18:1  (median [IQR]) | 5.470  [4.130, 7.415] | 5.363  [3.993, 7.660] | 5.480  [4.200, 7.255] | 0.712 |
| PG  (median [IQR]) | 8.464  [4.926, 13.333] | 7.900  [4.740, 12.768] | 8.676  [5.118, 13.642] | 0.900 |
| HexCER  (median [IQR]) | 2.931  [2.121, 3.812] | 2.733  [1.914, 3.737] | 3.039  [2.223, 3.815] | 0.970 |
| PS  (median [IQR]) | 9.427  [5.199, 18.315] | 9.672  [5.322, 18.826] | 9.174  [5.162, 18.027] | 0.970 |

^ Reported p-values are derived from t-tests and subsequently subjected to false discovery rate correction to account for multiple comparisons. Significance codes: * p < 0.0001, ** p < 0.00001, *​** p < 0.000001.

**Supplemental Table 4.** Shotgun Lipidomics was used to compare circulating levels of major classes of endogenous lipids by HYPO vs. NORMO subphenotype. Cholesterol Esters (CE), lysophosphatidylcholines (LPC), and sphingomyelins (SM) were significantly reduced in HYPO patients. All lipid levels are in nmoles/mL. The other classes of lipids analyzed were, Phosphatidic Acid (PA); Phosphatidylcholine (PC); Phosphatidylinositol (PI); Triacylglycerol (TG); Free Fatty Acids (FFA); Lactosylceramides (LacCER); Ceramides (Cer); Phosphatidylglycerol (PG); Hexosylceramides (HexCER); and Phosphatidylserine (PS); HYPO = Hypolipoprotein; NORMO = Normolipoprotein; IQR = Interquartile Range

**Supplemental Table 5. Shotgun Lipidomics Individual Lipids.**

| **Lipid** | **Class** | **Total Cohort**  **(n = 271)** | **HYPO Cohort**  **(n = 116)** | **NORMO Cohort**  **(n = 155)** | **Adjusted**  **P-value^** |
| --- | --- | --- | --- | --- | --- |
| SM d18:1/20:1  (median [IQR]) | SM | 3.801  [2.712, 4.900] | 3.190  [2.174, 3.807] | 4.570  [3.468, 5.380] | 0.000 |
| CE 18:2  (median [IQR]) | CE | 541.182  [340.787, 759.250] | 358.285  [220.713, 590.077] | 674.379  [478.628, 856.862] | 0.000 |
| CE 20:4  (median [IQR]) | CE | 134.211  [82.020, 186.211] | 87.630  [41.515, 149.039] | 158.814  [117.804, 216.782] | 0.000 |
| CE 18:1  (median [IQR]) | CE | 227.555  [163.270, 309.533] | 171.914  [110.434, 248.594] | 262.549  [204.629, 331.998] | 0.000 |
| CE 16:0  (median [IQR]) | CE | 160.266  [107.399, 213.994] | 122.478  [71.204, 173.389] | 189.959  [140.714, 234.653] | 0.000 |
| CE 22:6  (median [IQR]) | CE | 8.508  [4.988, 13.459] | 5.832  [2.992, 9.684] | 11.003  [6.809, 15.774] | 0.000 |
| CE 20:3  (median [IQR]) | CE | 8.326  [4.636, 12.697] | 5.628  [3.207, 8.809] | 10.372  [6.411, 14.525] | 0.000 |
| CE 18:0  (median [IQR]) | CE | 7.379  [4.556, 10.646] | 5.120  [3.590, 8.544] | 8.702  [5.897, 11.932] | 0.000 |
| LPC 16:0  (median [IQR]) | LPC | 13.578  [7.210, 20.629] | 8.909  [5.083, 15.344] | 16.850  [11.090, 23.492] | 0.000 |
| SM d18:1/18:0  (median [IQR]) | SM | 16.676  [11.987, 22.067] | 13.900  [9.374, 19.213] | 18.828  [14.850, 23.878] | 0.000 |
| SM d18:1/22:1  (median [IQR]) | SM | 15.679  [12.474, 19.863] | 13.677  [11.124, 16.994] | 17.558  [14.217, 21.459] | 0.000 |
| CE 18:3  (median [IQR]) | CE | 14.445  [8.521, 21.100] | 10.800  [6.778, 15.872] | 17.347  [11.252, 23.868] | 0.000 |
| SM d18:1/18:1  (median [IQR]) | SM | 8.183  [5.857, 10.876] | 6.581  [4.316, 9.365] | 9.380  [7.251, 12.037] | 0.000 |
| SM d18:1/24:0  (median [IQR]) | SM | 12.564  [9.815, 16.146] | 11.239  [8.642, 13.712] | 13.462  [11.076, 17.460] | 0.000 |
| PC 16:0_22:5  (median [IQR]) | PC | 17.193  [11.889, 23.244] | 13.721  [9.677, 18.814] | 19.447  [14.038, 25.558] | 0.000 |
| CE 20:5  (median [IQR]) | CE | 6.853  [3.913, 10.553] | 4.590  [2.420, 7.373] | 8.073  [5.472, 12.479] | 0.000 |
| CE 14:0  (median [IQR]) | CE | 4.475  [2.597, 6.600] | 3.930  [1.933, 5.050] | 5.498  [3.311, 7.352] | 0.000 |
| PC 16:0_22:6  (median [IQR]) | PC | 37.936  [26.594, 54.312] | 31.868  [20.037, 44.707] | 44.921  [30.778, 64.322] | 0.000 |
| PC 16:0_20:3  (median [IQR]) | PC | 34.059  [24.291, 47.714] | 29.376  [20.455, 39.317] | 38.389  [28.840, 51.291] | 0.000 |
| FA 24:1  (median [IQR]) | FA | 3.097  [2.524, 7.890] | 3.561  [2.621, 9.571] | 2.887  [2.485, 4.092] | 0.002 |
| PC 16:0_20:4  (median [IQR]) | PC | 162.994  [112.994, 214.855] | 140.746  [101.501, 185.853] | 182.793  [123.872, 233.977] | 0.002 |
| CE 16:1  (median [IQR]) | CE | 33.279  [21.460, 49.130] | 28.139  [18.185, 42.414] | 38.291  [25.981, 54.707] | 0.003 |
| PC 16:0_20:2  (median [IQR]) | PC | 2.660  [2.082, 3.468] | 2.399  [1.875, 3.013] | 2.954  [2.290, 3.719] | 0.003 |
| FA 24:0  (median [IQR]) | FA | 1.904  [1.623, 3.296] | 2.067  [1.647, 3.783] | 1.887  [1.612, 2.469] | 0.003 |
| PC 18:1_20:4  (median [IQR]) | PC | 11.518  [8.548, 15.376] | 10.150  [7.491, 12.737] | 12.608  [10.001, 16.507] | 0.004 |
| TG 57:10-FA22:6  (median [IQR]) | TG | 0.693  [0.598, 0.797] | 0.653  [0.587, 0.747] | 0.753  [0.611, 0.824] | 0.004 |
| PC 18:1_20:3  (median [IQR]) | PC | 2.538  [1.665, 3.404] | 2.301  [1.613, 3.007] | 2.673  [1.771, 3.732] | 0.007 |
| PA 34:4  (median [IQR]) | PA | 2.262  [2.202, 2.320] | 2.246  [2.168, 2.305] | 2.274  [2.227, 2.338] | 0.008 |
| LPC 18:2  (median [IQR]) | LPC | 3.899  [2.042, 6.657] | 2.628  [1.629, 4.962] | 4.708  [2.845, 8.005] | 0.009 |
| SM d18:1/24:1  (median [IQR]) | SM | 47.662  [38.972, 60.701] | 43.898  [36.769, 54.510] | 50.803  [41.734, 63.903] | 0.010 |
| SM d18:1/22:0  (median [IQR]) | SM | 25.271  [20.159, 33.335] | 22.665  [18.762, 28.127] | 27.101  [21.912, 34.359] | 0.021 |
| LPC 18:1  (median [IQR]) | LPC | 3.500  [2.024, 5.334] | 2.589  [1.533, 4.389] | 4.096  [2.468, 6.606] | 0.027 |
| PC 16:0_16:1  (median [IQR]) | PC | 8.780  [6.211, 13.326] | 10.442  [6.845, 15.810] | 7.881  [5.593, 12.299] | 0.032 |
| FA 22:2  (median [IQR]) | FA | 1.077  [0.883, 1.305] | 1.132  [0.943, 1.418] | 1.027  [0.853, 1.234] | 0.049 |

**Supplemental Table 5**. Analysis of individual lipid species and their respective classes between the HYPO and NORMO cohorts. It includes the specific lipid species identified, their classification, the average values for each cohort, and the statistical significance.

^ Reported p-values are derived from t-tests and subsequently subjected to false discovery rate correction to account for multiple comparisons.

Cholesterol Esters (CE); Sphingomyelins (SM); Lysophosphatidylcholines (LPC); Phosphatidic Acid (PA); Phosphatidylcholine (PC); Triacylglycerol (TG); Fatty Acids (FA); HYPO = Hypolipoprotein; NORMO = Normolipoprotein

**Supplemental Table 6. Lipid Panel Results**

| **Lipid** | **Total Cohort**  **(n = 257)** | **HYPO Cohort**  **(n = 111)** | **NORMO Cohort**  **(n = 146)** | **Adjusted**  **P-value^** |
| --- | --- | --- | --- | --- |
| 9HODE  (median [IQR]) | 1.746  [1.186, 2.557] | 1.777  [1.201, 2.643] | 1.691  [1.181, 2.523] | 0.4953 |
| 14S-HDHA*  (median [IQR]) | 0.466  [0.166, 0.899] | 0.354  [0.158, 0.886] | 0.487  [0.173, 0.896] | 0.4953 |
| 11HETE*  (median [IQR]) | 0.126  [0.060, 0.233] | 0.128  [0.066, 0.229] | 0.120  [0.057, 0.241] | 0.4953 |
| 12HETE  (median [IQR]) | 6.908  [1.868, 21.180] | 7.712  [1.668, 22.480] | 6.298  [2.122, 20.585] | 0.4953 |
| RVD1*  (median [IQR]) | 0.060  [0.038, 0.115] | 0.061  [0.037, 0.119] | 0.059  [0.038, 0.113] | 0.4963 |
| 13HODE  (median [IQR]) | 2.873  [2.123, 4.213] | 2.839  [1.997, 4.543] | 2.921  [2.147, 3.986] | 0.6056 |
| 5HETE  (median [IQR]) | 0.323  [0.184, 0.565] | 0.366  [0.196, 0.588] | 0.300  [0.178, 0.548] | 0.6056 |
| 15HETE  (median [IQR]) | 0.204  [0.140, 0.373] | 0.201  [0.138, 0.330] | 0.220  [0.143, 0.422] | 0.6243 |

**Supplemental Table 6.** Lipid mediators between the HYPO and NORMO cohorts, their concentrations in each cohort, and the statistical significance.

* Lipids with missing values.

^ Reported p-values are derived from t-tests conducted on multiple imputations, and subsequently subjected to false discovery rate correction to account for multiple comparisons.

Hydroxyoctadecadienoic acids (HODEs); 14(S)-hydroxy Docosahexaenoic Acid (14S-HDHA); Hydroxyeicosatetraenoic (HETEs); Resolvin D1 (RVD1); HYPO = Hypolipoprotein; NORMO = Normolipoprotein; IQR = Interquartile Range

Shotgun Lipidomics/Lipid Panel

The process of lipid extraction for shotgun lipidomic analysis has been detailed elsewhere.^1^ In brief, 25μl of plasma was placed into a glass tube for extraction, and a modified Bligh and Dyer extraction was conducted with an initial ratio of 0.9:2:1 (water:methanol:chloroform) and a final ratio of 1.9:2:1.9. Before the biphasic extraction, each sample received an internal standard mixture containing 70 lipid standards across 17 subclasses. Following two successive extractions, the pooled organic layers were dried down in a Thermo SpeedVac SPD300DDA using ramp setting 4 at 35 degrees Celsius for 45 minutes with a total run time of 90 minutes. The lipid samples were then resuspended in 300μl of 1:1 methanol/dichloromethane with 10mM Ammonium Acetate and transferred to robovials (Thermo 10800107) for analysis.

Samples were analyzed on the Sciex 5500^TM^ with DMS device, akin to the Sciex Lipidyzer^TM^ Platform (Framingham, MA) employing an expanded targeted acquisition list comprising 1450 lipid species across 17 subclasses. The Differential Mobility Device was tuned with EquiSPLASH LIPIDOMIX Avanti 330731 (Alabaster, AL). The instrument method, encompassing settings, tuning protocol, MRM list, and data analysis workflow, followed previously described procedures.^1^ Essentially, all tuning and data acquisition were executed in Analyst 1.7.1, and data analysis was conducted using the Shotgun Lipidomics Assistant application. Compensation voltages (COV) tuning for individual lipid classes were determined by ramping the compensation voltage while acquiring representative class standards, which were then used to update the acquisition method. The 1450 targeted lipid species were acquired over two infusions of 75μl each, with each targeted MRM acquired 20 times. The averaged raw signal was quantified against assigned standards and normalized to milliliters of plasma.

The LC-MS/MS method employed for the lipid panel has been previously outlined.^1,2^ In short, this method covers 39 distinct bioactive lipids, degradation products, and pathway markers of the cyclooxygenase and lipoxygenase products derived from arachidonic acid, linoleic acid, docosahexaenoic acid, or eicosapentaenoic acid. Each analyte was associated with one of 19 distinct structurally identical or class-specific deuterated internal standards. If an analyte lacked a structurally identical internal standard, an internal standard sharing the same basic structure and co-eluting within 0.5 minutes of the analyte in question was assigned.

Lipids were extracted from 100µl of plasma by mixing BHT (20μM), 50μl of 20 ng/ml internal standard, and 150μl of methanol. After vortexing and centrifugation at 15,000 RPM for 10 minutes at room temperature, the supernatant was collected and 1.8ml of HCl-acidified H2O (pH = 3-4) was added. Solid phase extraction was conducted using 3 cc Oasis HLB cartridges, with elution performed using 2ml of methanol. The eluate was then dried down under argon and reconstituted in 100μl of methanol for LCMS analysis. Chromatography was carried out on an Agilent 1290 UHPLC system using a Phenomenex Kinetex C18 column (2.6μM particle size, 2.1mm ID x 150mm) with gradient elution. Mass spectrographic analysis was performed on a SCIEX 5500 QTrap run in negative ion mode, and data analysis was conducted using MultiQuant software, with the concentration of each analyte being determined relative to its internal standard and against a standard curve. Final data was normalized to plasma volume.

External Data Set preparation and processing

External data sets were obtained from the sequence read archive (SRA). Data were processed as followed prior to analysis.

The study by Scicluna et al (GSE65682) is a microarray dataset. The matrix was directly downloaded from Refine.Bio, who quantile normalized the dataset with a reference distribution. Because these were microarray data, we were unable to reprocess the data. Ensembl genes were mapped to symbols using  org.Hs.eg.db library in R.

The study by Baghela et al (GSE185263) was an RNA-seq data set, which allowed us to obtain raw reads from SRA and reprocess them mimicking Refine.Bio's pipeline. This included aligning reads using Salmon and then using tximport to get the count matrix. Ensembl genes were then mapped to symbols using  org.Hs.eg.db library in R. tximport corrects for average transcript length so that a gene is not labeled differentially expressed due to transcript length.

REFERENCES

1. Sulaiman D, Wu D, Black LP, et al. Lipidomic changes in a novel sepsis outcome-based analysis reveals potent pro-inflammatory and pro-resolving signaling lipids. *Clin Transl Sci*. 2024;17(3):e13745. doi:10.1111/cts.13745

2. Meriwether D, Sulaiman D, Volpe C, et al. Apolipoprotein A-I mimetics mitigate intestinal inflammation in a COX2-dependent inflammatory disease model. *J Clin Invest*. 2019;129(9):3670-3685. doi:10.1172/JCI123700
